# Juvenile survival of little owls decreases with snow cover

**DOI:** 10.1002/ece3.11379

**Published:** 2024-05-20

**Authors:** Marco Perrig, Steffen Oppel, Matthias Tschumi, Herbert Keil, Beat Naef‐Daenzer, Martin U. Grüebler

**Affiliations:** ^1^ Swiss Ornithological Institute Sempach Switzerland; ^2^ Institute of Evolutionary Biology and Environmental Studies University of Zurich Zurich Switzerland; ^3^ Forschungsgemeinschaft zur Erhaltung einheimischer Eulen (FOGE) Oberriexingen Germany

**Keywords:** Bayesian mark–recapture, climate change, demography, extreme weather, natal dispersal, radio‐tracking, winter mortality

## Abstract

Global environmental changes are associated with warmer average temperatures and more extreme weather events, potentially affecting wildlife population dynamics by altering demographic processes. Extreme weather events can reduce food resources and survival in all seasons of the year. Estimates of season‐specific survival probabilities are therefore crucial to understand the moderating effect of extreme events on annual mortality. Here, we analysed survival probabilities of 307 radio‐tracked juvenile little owls (*Athene noctua*) over two‐week periods from fledging to their first breeding attempt in the following spring to assess the contribution of extreme weather events. Survival probabilities were typically lowest during the first weeks after fledging in summer but were moderated by seasonal extremes in winter. The duration of snow cover in winter had a strong negative effect on survival probability, while being food supplemented during the nestling stage increased survival during the first weeks after fledging in summer and ultimately led to a larger proportion of birds surviving the first year. Overall annual survival probability over the first year varied by 34.3% between 0.117 (95% credible interval 0.052–0.223) and 0.178 (0.097–0.293) depending on the severity of the winter, and was as high as 0.233 (0.127–0.373) for food‐supplemented fledglings. In years with mild winters, the season with the lowest survival was the summer post‐fledging period (0.508; 0.428–0.594), but in years with extensive snow cover the winter was the season with the lowest survival (0.481; 0.337–0.626). We therefore show that extreme weather events occurring in a particular season reduced the proportion of first‐year survivors. Increasing extreme weather events can moderate seasonal survival probability through altering food supply of juvenile little owls either during the nestling period or in winter, with similarly large effects on annual survival and the viability of populations.

## Graphical abstract

Climatic changes can affect the survival of young birds and populations. Using high‐resolution telemetry, we show that annual juvenile survival of little owls can be reduced by 34% during harsh winters. Warmer winters with less snow may therefore improve juvenile survival.

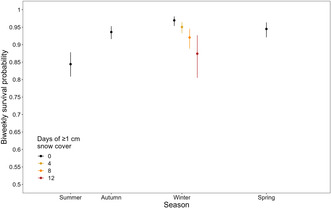

## 1 INTRODUCTION

Global environmental change is predicted to lead to warmer average temperatures and more extreme weather events (Stott, 2016). If these extreme weather events affect demographic processes, such as the reproduction, survival, or dispersal of individuals, environmental changes may contribute directly to wildlife population dynamics (Saracco & Rubenstein, 2020; Shriver, 2016). In birds, juvenile life stages are often critical to the growth rate of populations (Clark & Martin, 2007; Finkelstein et al., 2010; Newton, 1989; Robinson et al., 2004), and to understand the consequences of environmental change on populations, we require a better understanding to what extent extreme events affect juvenile survival.

The survival of juvenile birds from fledging to their first reproduction is generally lower than the survival of adults (Maness & Anderson, 2013; Naef‐Daenzer & Grüebler, 2016; Newton et al., 2016). Because juveniles are more susceptible to extreme weather events (Robinson et al., 2007), juvenile survival can vary enormously among years partly due to environmental conditions (Gaillard & Yoccoz, 2003; Harris et al., 2007; Souchay et al., 2013). However, extreme weather events are most likely to affect survival in particular seasons or life stages. Because global environmental change may lead to different extreme events in different seasons, it is critical to understand how environmental conditions affect season‐specific survival probability and how these seasonal effects moderate overall annual survival probability (Rushing et al., 2017).

The period from fledging to first reproduction in young birds includes several distinct life‐history stages such as the post‐fledging period in the natal home range, dispersal and migration, wintering, and habitat selection and settlement at the first breeding site. All these different stages involve distinct challenges and therefore may impose different constraints on juvenile survival (Buechley et al., 2021; Grande et al., 2009; Grüebler, Korner‐Nievergelt, & Naef‐Daenzer, 2014; Robinson et al., 2004; Ward et al., 2004). Environmental conditions are known to affect survival during certain life‐history stages more than others (Dybala et al., 2013; Maness & Anderson, 2013; Reid et al., 2008), and the effects of environmental changes on juvenile survival may therefore differ between the life‐history stages of the pre‐recruiting phase. To understand which life‐history stages are particularly important for population dynamics, and to predict the consequences of environmental changes on populations, the contribution of environmental variation in each life‐history stage to pre‐reproductive survival must be understood (Cox et al., 2014; Grüebler, Korner‐Nievergelt, & Naef‐Daenzer, 2014; Low & Pärt, 2009; Robinson et al., 2004).

The life‐history transition of fledging generally results in high mortality as fledged birds need to survive independently in unfamiliar environments (Cox et al., 2014; Low & Pärt, 2009; Naef‐Daenzer & Grüebler, 2016). Following that period, young birds departing from their natal site face two further significant challenges in consecutive life‐history stages. First, they move through and explore new, unfamiliar, and potentially inhospitable environments during natal dispersal (Bowler & Benton, 2005; Clobert et al., 2012; Low & Pärt, 2009; Robinson et al., 2004; Roque et al., 2021; Stillman et al., 2021). Second, they face the reduced availability and accessibility of food, and simultaneously increased thermoregulatory costs during winter (Altwegg et al., 2006; Rubáčová et al., 2021; Thorup et al., 2013). However, whether mortality is mainly associated with the post‐fledging and dispersal phases, or with the environmentally challenging period during winter is poorly studied and may be moderated by the occurrence of extreme events (Dybala et al., 2013; Grüebler, Korner‐Nievergelt, & Naef‐Daenzer, 2014; Rushing et al., 2017).

Here, we examined the first‐year survival of little owls (*Athene noctua*) at two‐week temporal resolution to determine season‐specific survival probabilities from fledging to the first reproductive attempt. The little owl is a small generalist mesopredator, inhabiting various open landscapes of Europe and Asia (Glue & Scott, 1980). Many populations of little owls in central Europe have decreased in recent decades, and harsh winters with extended periods of snow cover have resulted in occasional population collapses (Van Nieuwenhuyse et al., 2023). Demographic analyses have indicated that juvenile survival and immigration are key demographic factors explaining differences in population growth rates (Le Gouar et al., 2011; Schaub et al., 2006). Therefore, it is important to understand the critical bottlenecks in the first year of the little owls' life (Thorup et al., 2013; Tschumi et al., 2019) and to identify the environmental factors affecting survival in different juvenile life‐history stages (Le Gouar et al., 2011; Perrig et al., 2014; Thorup et al., 2010). We have previously shown that survival of juvenile little owls was very low just after fledging, varied with fledgling body condition associated with nestling food supply, and increased over the first 2 months post‐fledging (Perrig et al., 2017). However, it is unclear whether the subsequent dispersal and wintering stages impose an equal or different toll on the survival of juvenile little owls, and what effect extreme winter weather events have on the number of little owls surviving the first year.

In this study, we investigated two main hypotheses considering the period between independence and the first breeding season. First, we predicted that survival during autumn, when juveniles dispersed from parental territories, would be lower than in winter and the following spring because of the risk of exploring unfamiliar environments. Second, we predicted that survival during winter would be reduced depending on the severity of winter conditions because of limited access to food resources (Altwegg et al., 2006; Le Gouar et al., 2011; Rubáčová et al., 2021). We estimated survival probabilities from the post‐fledging period to the first breeding season and could thus identify how environmental conditions determine the most important seasonal bottleneck within the first year of juvenile little owls. This information will be critical to understand the potential future effects of a changing climate on population dynamics of little owls.

## 2 METHODS

### 2.1 Study population

The study was conducted in southwestern Germany (Landkreis Ludwigsburg, Baden‐Württemberg, 48°53′43″ N, 9°11′45″ E) in an area of ~700 km^2^ at elevations ranging from 120 to 250 m above sea level. The landscape in the study area is composed of intensively used agricultural fields, meadows, orchards, and vineyards (56%), forests (25%), and urban settlements (17%), containing a high density and diversity of small structural elements and management regimes (Fattebert et al., 2018, 2019; Hauenstein et al., 2019; Perrig et al., 2014; Tschumi et al., 2020).

The little owl is a small (160–250 g) nocturnal bird breeding in tree cavities and nest boxes in orchards of traditional agricultural landscapes (Van Nieuwenhuyse et al., 2023). The breeding season of the species in Germany lasts from April until July, with the peak time of fledging in June. Fledglings disperse from their natal territory usually within 65 days after fledging (Fattebert et al., 2019; Perrig et al., 2017). First reproduction normally occurs at the age of 1 year, following the initial dispersal from the natal territory, and a stationary period during winter (Exo, 1992; Hauenstein et al., 2019). Survival of adults and the quality of fledged offspring are strongly linked to habitat quality and food availability (Michel et al., 2022; Perrig et al., 2014; Schaub et al., 2006; Thorup et al., 2010).

### 2.2 Monitoring survival

From 2009 to 2011, 93 broods were closely monitored from egg laying until fledging by conducting regular brood controls and using camera traps (Perrig et al., 2014, 2017). Prior to fledging, at an average age of 28.7 days (± 2.93 standard deviation), all 307 chicks of these broods were tagged with VHF radio transmitters (Naef‐Daenzer et al., 2005) using a standard backpack harness configuration (Kenward et al., 2001). The total tag mass (including harness) was 6.9–7.2 g (3%–5% of the birds' body mass), and similar transmitter attachments had no measurable adverse effect on the survival probability of little owls (Sunde et al., 2014; Thorup et al., 2013) or smaller songbirds (Naef‐Daenzer et al., 2001a, 2001b). The expected lifespan of the battery was 400 days, and the detection range of the VHF signals was up to 40 km (for details, see Perrig et al., 2017).

We used handheld antennas to locate all individuals at least three times per week throughout the study period from May 2009 until May 2012, except during four 2‐week intervals in winter 2009 and early spring 2010 when no radio‐tracking could be carried out. In an additional six 2‐week intervals (mid‐winter 2009 and late winter 2010), the tracking effort was reduced, and individuals were located less than three times per week.

### 2.3 Temporal, individual, and environmental variables affecting survival

To quantify seasonal variation in survival, we defined four discrete seasons by date that correspond to differences in environmental conditions and the typical behaviour of juvenile birds: (1) summer (15 May–1 August): the post‐fledging phase after juveniles first leave the nest (previously analysed in Perrig et al., 2017); (2) autumn (2 August–23 October): the dispersal period when individuals permanently depart from their natal territory, and environmental conditions change to shorter days and cooler temperatures; (3) winter (24 October–12 March): the period when birds establish and occupy a winter home range, and endure occasionally cold winter weather during which food can become inaccessible; (4) spring (13 March–15 June): the first breeding period when birds acquire and occupy the first territory and breed during gradually warming weather with longer day lengths. Note that our season definition includes a deliberate overlap of 4 weeks between subsequent years to accommodate the staggered fledging date of juveniles and staggered onset of first reproduction.

Besides temporal variation, survival may also differ by age, sex, and body mass (Le Gouar et al., 2011; Tschumi et al., 2019). We therefore measured body mass (to the nearest 0.1 g) and tarsus length (to the nearest 0.1 mm) on the day of tagging, and corrected these measurements for the age of the bird at the time of measurement (Perrig et al., 2014) to account for older chicks generally being larger. We specified the age of juveniles in days based on standard pictures and the known fledging date (Perrig et al., 2017). In addition, feather samples were obtained for genetic sex determination of the nestlings (Tschumi et al., 2019). In 2010 and 2011, roughly half of the monitored broods were provided with supplementary food during the nestling stage (Perrig et al., 2017), and we recorded whether individuals had benefitted from supplementary feeding or not.

To investigate the effect of winter conditions on survival probabilities, we extracted daily snow depths from the weather station Sachsenheim (Germany, station ID: 04349, downloaded from: https://opendata.dwd.de/climate_environment/CDC/observations_germany/climate/daily/kl/historical/, accessed 16 Oct 2023). Snow depth is known to limit owl's access to food and is therefore a primary indicator of winter harshness and food availability that would influence survival (Altwegg et al., 2006; Le Gouar et al., 2011; Thorup et al., 2013). We calculated the number of days with a snow cover ≥1 cm for each two‐week encounter occasion (alternative measures of larger snow depths did not affect our conclusions).

### 2.4 Estimating survival probabilities

Radio‐tracking data were aggregated into encounter histories of two‐week intervals, which we refer to as ‘biweekly’. The encounter histories were used in a Cormack–Jolly–Seber (CJS) mark–recapture model estimating biweekly survival while controlling for variation in detection probability and individual variability (Kéry & Schaub, 2012; Lebreton et al., 1992).

Because our key interest was to quantify seasonal differences in survival, our survival model included a fixed intercept for each of the four seasons (summer, autumn, winter, spring), as well as fixed effects to account for the mass and sex of each fledgling (Tschumi et al., 2019), and whether the fledglings came from broods that were provided with supplementary food (Perrig et al., 2017). We also included a fixed effect that explored whether duration of snow cover could explain variation in survival. Because body mass and size were highly correlated, and the variables age and body size did not affect survival in preliminary explorations, we retained the most parsimonious combination of variables (Hooten & Hobbs, 2015), and neither age nor size was retained in our survival model. We fitted three models to include the variables body mass and supplementary feeding in three alternative model formulations: either by affecting survival only in the immediate post‐fledging period (Perrig et al., 2017) or by allowing body mass and supplementary feeding to affect survival in every season over the first year of life (Catitti et al., 2022; Mainwaring et al., 2023; Nägeli et al., 2022).

To estimate detection probability, we included temporal variation in tracking effort as an explanatory variable due to the unequal tracking efforts across years and tracking periods. We specified that detection probability was zero during four intervals when no tracking effort occurred. For the remaining intervals, we estimated two distinct detection probabilities, one for those six biweekly periods with reduced effort in winter 2009 and 2010, and another for the remaining 80 periods with full tracking effort. We also included a random individual effect to account for residual variability in detection probability among individuals. Because severe winter weather may not only affect survival but may also lead to temporary escape movements to more benign areas (Gura, 2023; Mysterud, 2016; Sonerud, 1986), we included the same snow cover variable that we assumed to affect survival also for detection probability to account for possible temporary emigration and low detection probability during severe winter weather.

We used a Bayesian approach for inference to include existing prior information on the survival probability of little owls (Le Gouar et al., 2011; Schaub et al., 2006; Thorup et al., 2013). We fit the models in software JAGS v. 3.3 (Plummer, 2012) called from R 4.1.3 (R Core Team, 2023) via the ‘runjags’ library (Denwood, 2016). We used a mildly informative prior for the biweekly survival probability (beta distribution with *α* = 95 and *β* = 10) given previous information on little owl survival (Le Gouar et al., 2011; Thorup et al., 2013), and a similarly informative prior for the detection probabilities during occasions with normal (random uniform 0.7–1) and reduced effort (random uniform 0.3–0.9). We used vague normally distributed priors for all other parameters (mean = 0 and standard deviation = 1–2, see code in Appendix S1 for detailed priors of all parameters), and conducted a prior sensibility test to ensure that biologically plausible survival estimates resulted from our prior distributions (Banner et al., 2020). We ran three Markov chains for 3500 iterations each, discarded the first 200 iterations, and used every sixth iteration for inference. Convergence of the three chains for all monitored parameters was visually inspected using trace plots and tested using the Gelman–Rubin diagnostic (Brooks & Gelman, 1998) to confirm that all parameters had an R‐hat of <1.02. We implemented posterior predictive checks to assess the goodness‐of‐fit of the survival model (Conn et al., 2018; Gelman et al., 1996; Kéry & Schaub, 2012) and confirmed that there was no evidence for a lack of fit (Bayesian *p*‐value = .427). Code to replicate these analyses can be found at https://github.com/Vogelwarte/LittleOwlSurvival and in Supporting Information.

We present median parameter estimates (*β*) for covariates on the logit scale with 95% credible intervals. We also present posterior estimates of biweekly survival probability with 95% credible intervals for each of the four seasons based on birds of average body mass that did not receive supplementary food as nestlings. To facilitate interpretation and comparison with other survival estimates, we calculated season‐specific survival by raising biweekly survival to the power of the length of each season (summer: 4 periods, autumn: 6 periods, winter: 10 periods, spring: 6 periods). To predict survival in severe winters, we used the maximum length of intense snow cover periods during our study to decompose the 10 winter periods into two periods with extreme snow cover, three periods each with high and intermediate snow cover, and two periods without snow cover (resulting in 43% of 140 winter days experiencing snow cover), and we multiplied the respective survival probabilities to estimate overwinter survival. To estimate annual survival, we multiplied the four seasonal survival probabilities, which represents the annual survival probability from 1 July to 30 June of the following year. To visualise what proportion of juveniles survived over the first year of life, we simulated the proportion of 100 juveniles of average body mass that survived 26 biweekly periods from one summer to the next by multiplying the number of live birds by the two‐week‐specific survival probability. We present this proportion for four scenarios, namely for birds that did and did not receive supplementary food as nestlings during either a mild or a harsh winter.

## 3 RESULTS

**FIGURE 1:**
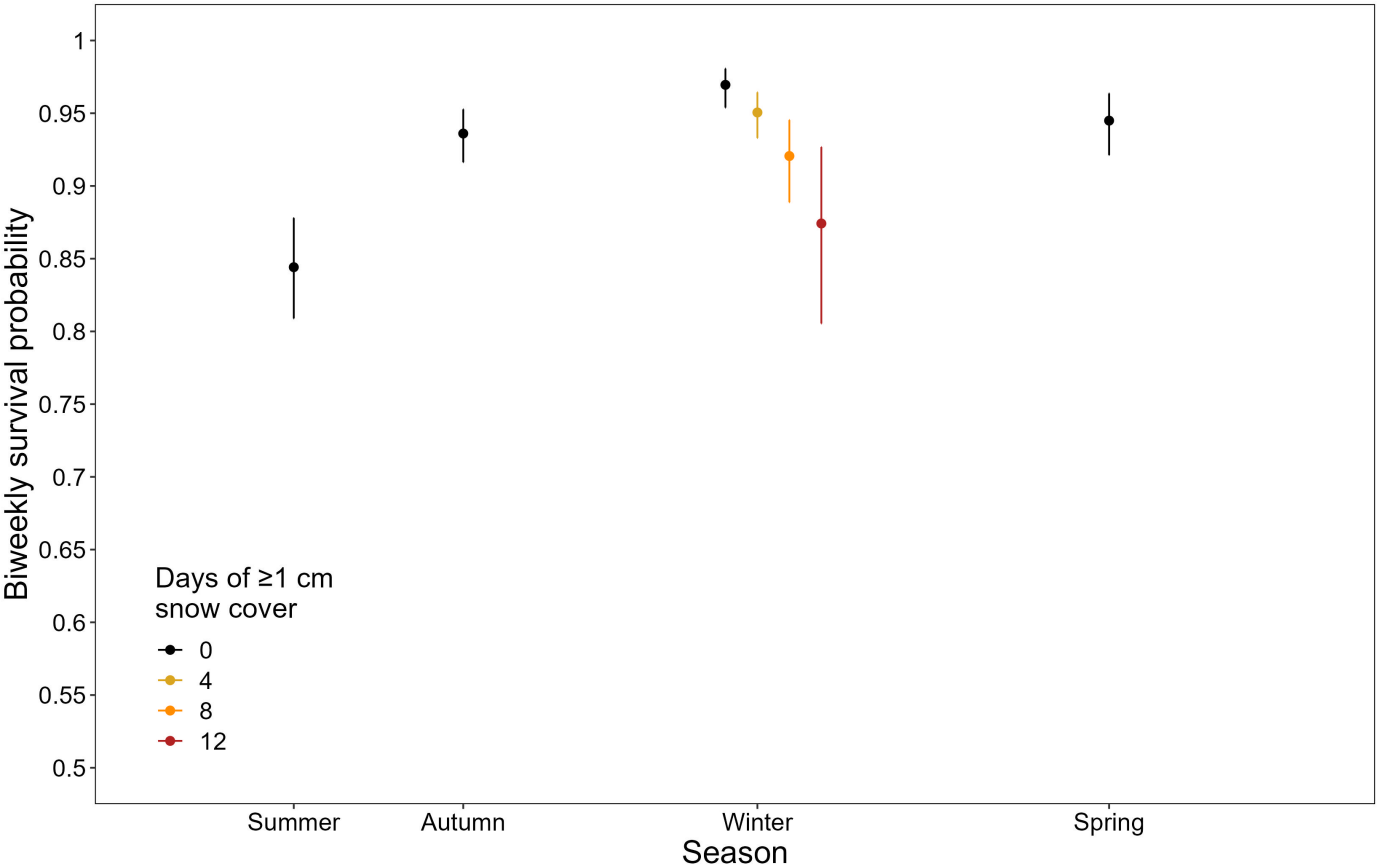
Estimated median survival probabilities of juvenile little owls of average body mass during the four seasons across their first year of life. Survival probability is scaled to biweekly encounter occasions, and therefore comparable across seasons despite the different durations of each season. Error bars represent 95% credible intervals.

Of the 307 individuals who left their nest, 46 (15%) survived to the end of the brood‐rearing stage in the following year. Our survival model estimated that biweekly survival was lowest in the summer post‐fledging season (0.844; 0.809–0.878), and substantially higher in autumn (0.936; 0.916–0.953), snow‐free winter (0.970; 0.954–0.981), and the following spring (0.945; 0.921–0.964; Figure 1) seasons. Accounting for the different durations of the seasons, the overall survival was lowest for the summer post‐fledging season despite its short duration, with the autumn dispersal season having marginally lower survival than a mild snow‐free winter and the following spring (Table 1).

**TABLE 1:** Estimated survival probabilities of juvenile little owls of average body mass in southwestern Germany during the four seasons of their first year, and cumulative annual survival for years with either mild or harsh winters.

Season	Duration (weeks)	Survival probability
Summer (post‐fledging)	8	0.508 (0.428–0.594)
Autumn	12	0.673 (0.592–0.748)
Winter (mild)	20	0.734 (0.624–0.823)
Winter (harsh)	20	0.481 (0.337–0.626)
Spring	12	0.712 (0.611–0.801)
Annual survival (with mild winter)	52	0.178 (0.097–0.293)
Annual survival (with harsh winter)	52	0.117 (0.052–0.223)

*Note*: Mild winter refers to winters without snow cover, harsh winter refers to 60 winter days experiencing snow cover of ≥1 cm.

**FIGURE 2:**
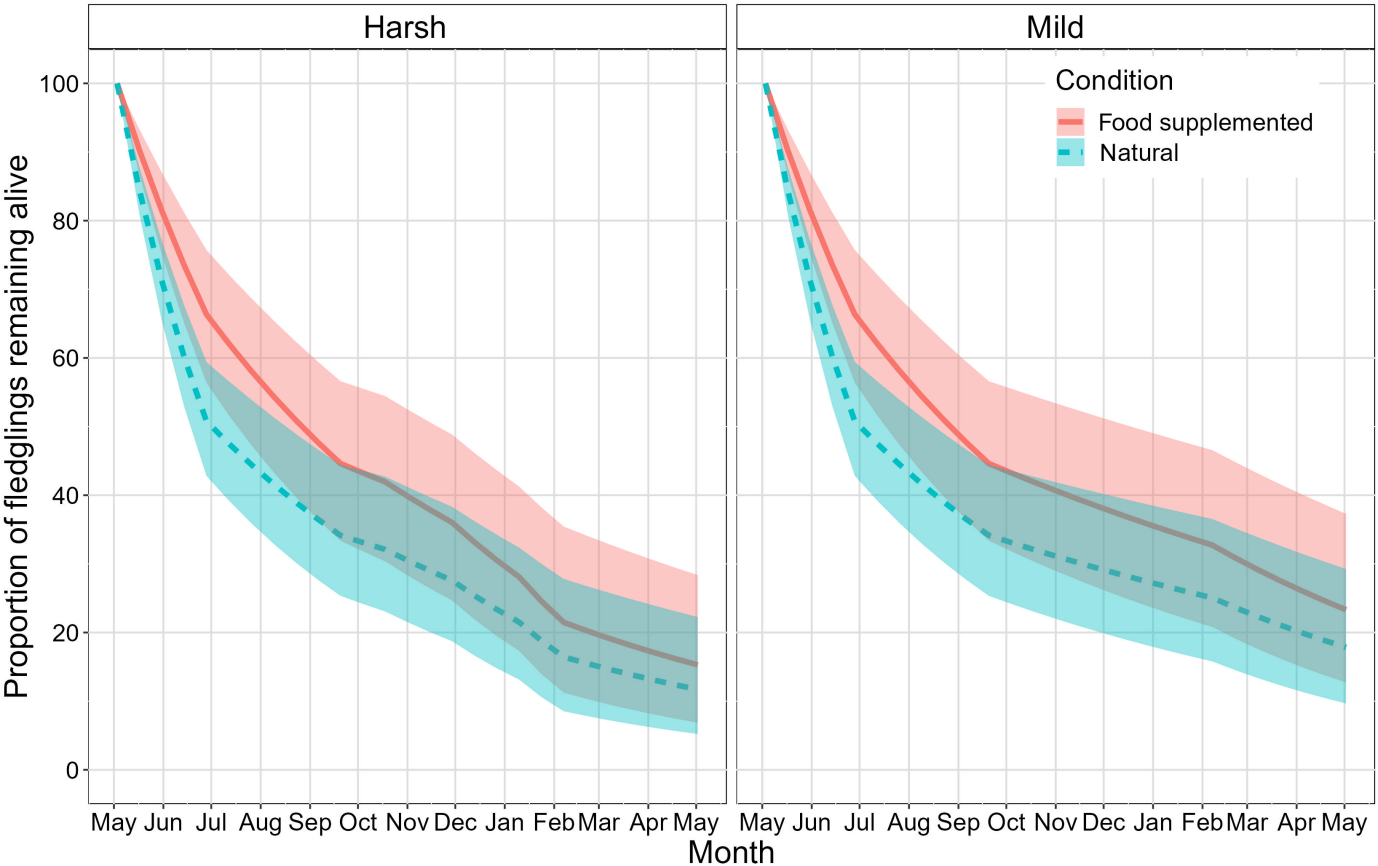
Estimated median proportion (and 95% confidence intervals) of juvenile little owls surviving from fledging in May until the first breeding season in the following year depending on the harshness of winter (left panel: harsh winter with 60 days of snow cover, right panel: mild winter with no snow cover), and whether they received supplementary feeding as nestlings (blue: unfed natural nestlings; red: nestlings provided with supplementary food).

Biweekly survival in winter decreased strongly with an increasing duration of snow cover (*β* = −0.389; 95% credible interval −0.571 to 0.201; Figure 1, Figure S1). By contrast, we found only a weak negative effect of snow cover on detection probability (*β* = −0.229; −0.468 to 0.022; Figure S1), and detection probability was very high (in periods with normal effort: 0.994; 0.989–0.996; in periods with reduced effort: 0.803; 0.625–0.892). In winters with extreme snow cover, biweekly survival decreased from 0.971 to 0.879 (Figure 1), and the survival over an entire harsh winter season was therefore even lower than during the post‐fledging summer (0.481; Table 1). In total, the annual survival probability of little owls during their first year of life ranged from 0.117 to 0.178 depending on the severity of the winter (Table 1). Thus, in years with long periods of snow cover, the winter period reduced first‐year survival by 34.3% compared to a snow‐free winter (Figure 2).

Besides the effect of snow cover, we also found evidence that survival increased with body mass (*β* = 0.480; 0.290–0.670), and with supplementary food provided to nestlings (*β* = 0.345; −0.073 to 0.758), but the latter effect was uncertain. Survival was only marginally higher for males than for females (*β* = 0.153; −0.256 to 0.521; Figure S1). We found that the effects of body mass (Figure S2) and food supplementation (Figure S3) on survival only occurred during the post‐fledging period but not later in the first year of juvenile little owls. However, those differences resulted in a 23.5% lower proportion of juveniles surviving the first year if they did not receive supplementary food (annual survival 0.233; 0.127–0.373), solely due to the difference in post‐fledging survival (Figure 2). Out of all the juveniles that died during the first year, the greatest proportion (39.7%–59.9%) perished during the post‐fledging period, while even harsh winters accounted for only 11.1%–27.3% of annual mortality (Table 2). Extrapolated seasonal survival estimates for birds of specific mass, sex, and feeding status are provided in Table S1.

**TABLE 2:** Seasonal contribution to the overall annual mortality over the first year of life of little owls.

Season	Natural		Supplementary fed	
	Mild winter	Harsh winter	Mild winter	Harsh winter
Summer	59.9	55.7	43.9	39.7
Autumn	20.2	18.8	28.3	25.6
Winter	11.1	20.1	15.5	27.3
Spring	8.8	5.4	12.3	7.3

*Note*: Numbers represent the proportion of juveniles that died in a given season out of all birds that died during the first year for mild and harsh winters and for birds that received supplementary food as nestlings or not. Mild winter refers to winters without snow cover, harsh winter refers to 60 winter days experiencing snow cover of ≥1 cm.

## 4 DISCUSSION

We show that winter survival in juvenile little owls was highly dependent on the number of days with snow cover, but that annual survival in years with mild winters was characterised by remarkably equal survival probabilities throughout seasons once individuals had survived the first weeks after fledging. Overall, only a fifth of juvenile little owls survived to the age of 1 year, and harsh winters with extended periods of snow cover reduced annual survival probability by >30%. However, the most important mortality bottleneck was the month after fledging, which accounted for 40%–60% of the total first‐year mortality. Supplementary food provided to nestlings increased annual survival by 23.5% due to its beneficial effect on post‐fledging survival. We therefore show that overall annual survival can be mediated by season‐specific survival influenced by extreme events and that events in different seasons may have similarly large effects on the first‐year survival of juvenile little owls. Because little owls are typical generalist predators that require access to small rodents and invertebrates as prime food source, the moderating effect of extreme weather events on annual survival is likely to be representative for many other species (Sonerud, 1986; but see Pavón‐Jordán et al., 2013; Rubáčová et al., 2021).

Our results are consistent with previous findings that harsh winters affect survival of little owls (Le Gouar et al., 2011; Michel, 2016; Thorup et al., 2013), but our study was able to disentangle mortality during the post‐fledging period in summer, during dispersal in autumn, and in winter. We show that winters with constant snow cover over 2 months result in less than half of juveniles surviving a winter (Table 1). Snow cover and frosty conditions are widely known to prevent owls and other resident bird species from accessing food, thus leading to reduced body condition and increased foraging efforts, which ultimately result in increased mortality rates (Altwegg et al., 2006; Kostrzewa & Kostrzewa, 1991; Naef‐Daenzer & Grüebler, 2016; Riegert & Fuchs, 2011; Sonerud, 1986). Young owls may also become more vulnerable to predators when they struggle to find suitable shelters and suffer from excessive thermoregulation costs (Bock et al., 2013; Grüebler, Widmer, et al., 2014; Naef‐Daenzer & Grüebler, 2016). As juvenile survival represents a key factor in little owl population dynamics (Le Gouar et al., 2011; Schaub et al., 2006), our results suggest strong population effects of extended periods of snow cover in this species, which is consistent with past evidence of population collapses after harsh winters from the last century (summarised in Van Nieuwenhuyse et al., 2023).

We found that survival increased for food supplemented and for heavier birds, but these effects occurred only in the post‐fledging period and were not detectable in subsequent seasons. A previous study revealed that early‐life effects associated with nestling food supplementation affected departure decisions from the parental home range, but not movement decisions during natal dispersal after departure (Fattebert et al., 2019). Similarly, early‐life conditions affected survival primarily during the post‐fledging period, but not afterwards, and we speculate that the selection imposed by early post‐fledging mortality reduces the influence of early‐life conditions at later stages in life (Sergio et al., 2014, 2019). Nonetheless, given the magnitude of the difference in survival between food‐supplemented and un‐supplemented (natural) little owl fledglings during the post‐fledging period, 23.5% more food‐supplemented fledglings survived the first year (Figure 2). Thus, favourable natal conditions in our study species may affect population‐level processes mainly by non‐random selection of juveniles in the nestling and post‐fledging period, with an effect of similar magnitude as that imposed by harsh winters.

Increased predation risk of inexperienced birds is widely known as a main factor for lower juvenile than adult survival probability during the first year of life in general (Clutton‐Brock et al., 1985; Maness & Anderson, 2013; Naef‐Daenzer & Grüebler, 2016; Sunde, 2005) and in little owls in particular (Naef‐Daenzer et al., 2017; Šálek et al., 2019). Juvenile predation rate is often further increased under poor food conditions (Coles & Petty, 1997; Rohner & Hunter, 1996) and when juveniles explore unknown areas (Bélichon et al., 1996; Lima, 1998; Yoder et al., 2004). Elevated costs during natal dispersal have led to the theory that the dispersal stage is a bottleneck with respect to survival and evolutionary ecology (Bartoń et al., 2012; Benard & McCauley, 2008; Bonte et al., 2012; Bowler & Benton, 2005; Hardouin et al., 2012; Väli et al., 2021). Our results show that in little owls the survival during the autumn dispersal season was not noticeably reduced. However, because we focused on a fixed temporal definition of the autumnal dispersal season, we were not able to investigate survival during the actual dispersal movement of individuals. Main natal dispersal movements are generally of short duration in little owls (median = 10 days; Fattebert et al., 2019). Mortality might be considerably increased during the few days of active dispersal without affecting our overall survival probability of the season. More detailed investigations of survival in relation to individual movements may reveal more nuanced patterns in survival probability and will illuminate the costs of dispersal in little owls (Yoder et al., 2004). Nestling food supply and its effects on post‐fledging survival may therefore be a more important factor for juvenile survival and the growth rate of populations than the dispersal or wintering stages (Cox et al., 2014; Grüebler, Korner‐Nievergelt, & Naef‐Daenzer, 2014; Low & Pärt, 2009; Martin et al., 2018; Naef‐Daenzer & Grüebler, 2016).

We found only a relatively small and uncertain effect of increased snow cover on detection probability, which was generally very high in our study. Thus, while some temporary emigration is possible and accounted for by our model, we can be confident that our estimate of lower survival in harsh winters is not affected by little owls temporarily leaving the study area during periods with extensive snow cover. We cannot exclude the possibility that little owls performed permanent escape movements that have been recorded in other owl species during severe winter conditions (Gura, 2023; Mysterud, 2016; Sonerud, 1986), because permanent emigration out of the monitored area and mortality are confounded in our data set (Naef‐Daenzer et al., 2017). However, permanent emigration would be most likely during the autumn dispersal phase (Hauenstein et al., 2019; Van Nieuwenhuyse et al., 2023), but we did not find a reduction in apparent survival during this season with the highest mobility of juveniles. We therefore suggest that permanent emigration likely only accounts for a small proportion of the estimated mortality.

Another potential confounding factor for our survival estimation is the burden of tagging which may affect the performance of individual birds (Bodey et al., 2018; Geen et al., 2019). Food‐supplemented and therefore heavier birds may have survived the post‐fledging period better because the weight of the transmitter could have affected these birds to a lesser degree than un‐supplemented and therefore lighter birds (Gervais et al., 2006), but lower post‐fledging survival is well known for individuals in poorer condition even when accounting for tag effects (Naef‐Daenzer et al., 2001a, 2001b; Naef‐Daenzer & Grüebler, 2016). In addition, survival probability of individuals with a harness‐mounted radio tag similar to the one we used here was not lower than survival of individuals equipped only with rings in studies of little owls and smaller songbirds (Naef‐Daenzer et al., 2001a, 2001b; Naef‐Daenzer & Grüebler, 2014; Sunde et al., 2014; Thorup et al., 2013). We therefore assume that transmitter attachment did not materially affect the survival probability of little owls, but we acknowledge that our study did not have an unmarked control group that would have allowed us to validate any potential effects of tag attachment on survival.

In summary, the survival of little owls during the first year can be characterised by two bottlenecks, differing in the underlying mechanisms. Juveniles first encounter a survival bottleneck in summer immediately after fledging from the nest, and another bottleneck in winter if environmental conditions reduce the accessibility of food. Contrary to the general hypothesis of elevated costs during natal dispersal, our results indicate that the autumn dispersal season in little owls is not more hazardous than other seasons during the first year of life. While increasingly warmer winters with less snow cover will likely have beneficial effects on juvenile survival probability and population dynamics of little owls, our study demonstrates the importance of understanding how overall survival is mediated by environmental conditions affecting season‐specific survival probabilities.

## AUTHOR CONTRIBUTIONS

**Marco Perrig:** Conceptualization (equal); data curation (equal); formal analysis (lead); methodology (lead); software (equal); writing – original draft (lead); writing – review and editing (equal). **Steffen Oppel:** Formal analysis (equal); writing – review and editing (equal). **Matthias Tschumi:** Data curation (equal); investigation (equal); project administration (equal); visualization (supporting); writing – review and editing (equal). **Herbert Keil:** Conceptualization (equal); funding acquisition (supporting); investigation (equal); methodology (supporting); project administration (equal); validation (equal); writing – review and editing (supporting). **Beat Naef‐Daenzer:** Conceptualization (lead); funding acquisition (equal); investigation (equal); methodology (equal); project administration (lead); resources (equal); supervision (lead); writing – original draft (equal); writing – review and editing (equal). **Martin U. Grüebler:** Conceptualization (equal); formal analysis (supporting); funding acquisition (lead); investigation (equal); methodology (equal); project administration (equal); resources (equal); supervision (equal); writing – original draft (equal); writing – review and editing (equal).

## CONFLICT OF INTEREST STATEMENT

The authors declare no competing interests.

## Supporting information


Appendix S1.

## Data Availability

The data and R code that support the findings of this study are openly available in GitHub (https://doi.org/10.5281/zenodo.10714914).
