# Supplementary material for: Juvenile survival of little owls decreases with snow cover
Source: Ecol Evol. 2024 May 20;14(5):e11379. doi: 10.1002/ece3.11379 (PMC11103642; doi:10.1002/ece3.11379)
Supplement: Supplementary file 1 — Appendix S1. [file ECE3-14-e11379-s001.docx]

**Supplementary Material**


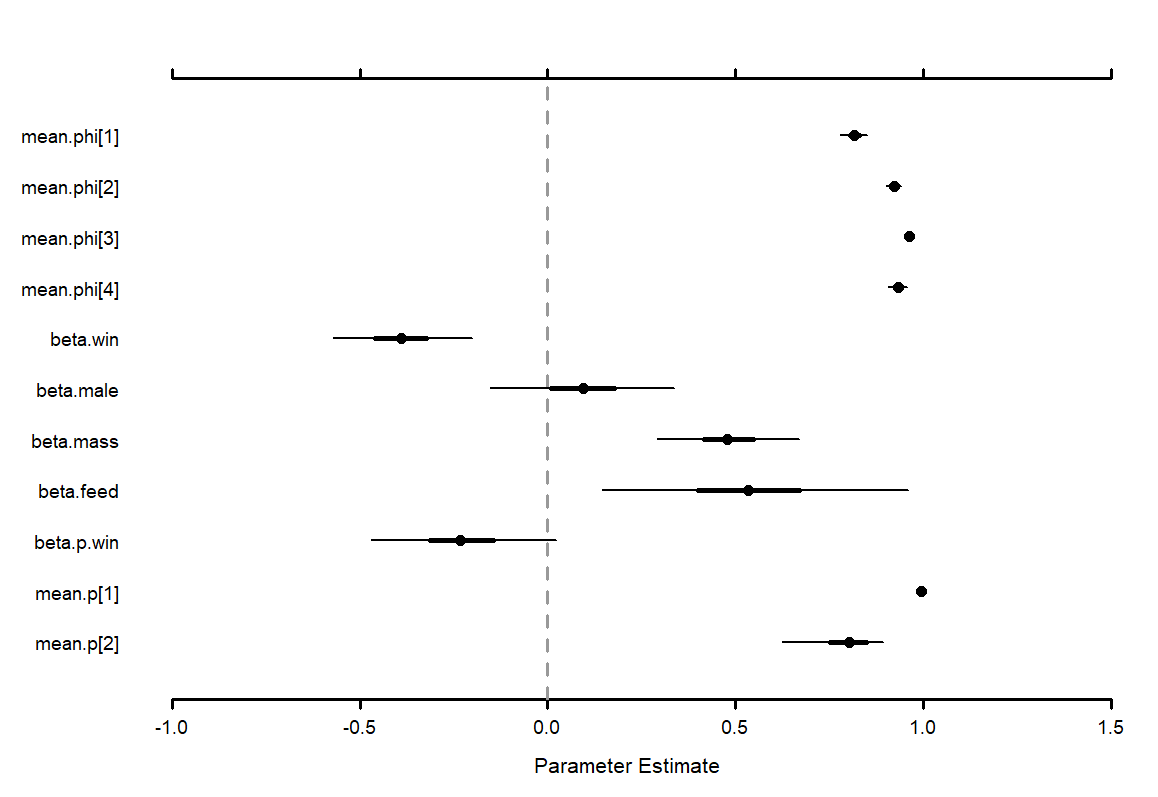


Fig. S1: Parameter estimates of the Cormack-Jolly-Seber model estimating biweekly survival and detection probability of radio-tracked little owls in Germany between 2009 – 2011. Mean.phi are survival probability intercepts for summer [1], autumn [2], winter [3], and spring [4], mean.p are detection probability intercepts for periods with full effort [1] and reduced effort [2], and ‘beta’ are parameter estimates for the linear predictors of winter snow cover (win), sex (male), body mass (mass), and supplementary feeding during the nestling phase (feed) on survival probability, and for the linear predictor of winter snow cover on detection probability (p.win).


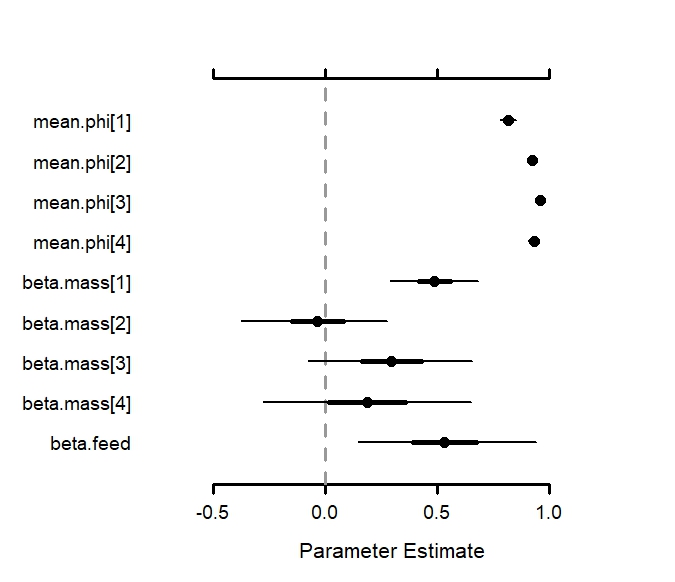


Fig. S2: Parameter estimates of a Cormack-Jolly-Seber model estimating biweekly survival and detection probability of radio-tracked little owls in Germany between 2009 – 2011, assuming that body mass affected survival in all seasons. Mean.phi are survival probability intercepts for summer [1], autumn [2], winter [3], and spring [4], and ‘beta’ are parameter estimates for the linear predictors of body mass (mass, for summer [1], autumn [2], winter [3], and spring [4]), and supplementary feeding during the nestling phase (feed) on survival probability.


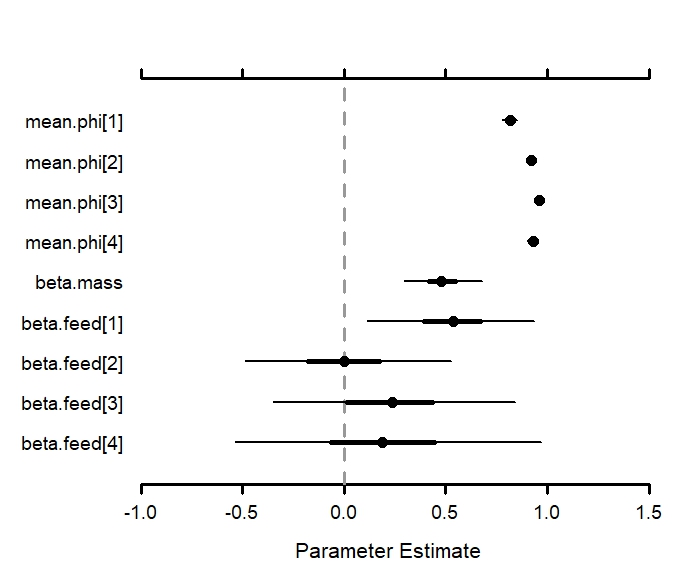


Fig. S3: Parameter estimates of a Cormack-Jolly-Seber model estimating biweekly survival and detection probability of radio-tracked little owls in Germany between 2009 – 2011, assuming that supplementary feeding affected survival in all seasons. Mean.phi are survival probability intercepts for summer [1], autumn [2], winter [3], and spring [4], and ‘beta’ are parameter estimates for the linear predictors of body mass (mass), and supplementary feeding during the nestling phase (feed, , for summer [1], autumn [2], winter [3], and spring [4]) on survival probability.

Table S1: Seasonal survival probabilities of radio-tracked little owls in Germany given as the median and 95% credible interval of survival estimates derived from a Cormack-Jolly-Seber model. Because the model accounted for sex, size, winter severity and supplementary feeding, survival estimates are provided for all possible parameter combinations. Food supplementation was provided during the chick-rearing period only, for details see (Perrig *et al.* 2017)

| Food Suppl. | Sex | Mass | Season | Survival probability (mild winter) | Survival probability (harsh winter) |
| --- | --- | --- | --- | --- | --- |
| No | Female | light | Summer | 0.196 (0.109 - 0.317) | |
| No | Female | light | Autumn | 0.661 (0.586 - 0.732) | |
| No | Female | light | Winter | 0.723 (0.615 - 0.812) | 0.465 (0.324 - 0.608) |
| No | Female | light | Spring | 0.701 (0.601 - 0.789) | |
| No | Female | average | Summer | 0.494 (0.421 - 0.567) | |
| No | Female | average | Autumn | 0.661 (0.586 - 0.732) | |
| No | Female | average | Winter | 0.723 (0.615 - 0.812) | 0.465 (0.324 - 0.608) |
| No | Female | average | Spring | 0.701 (0.601 - 0.789) | |
| No | Female | heavy | Summer | 0.752 (0.636 - 0.838) | |
| No | Female | heavy | Autumn | 0.661 (0.586 - 0.732) | |
| No | Female | heavy | Winter | 0.723 (0.615 - 0.812) | 0.465 (0.324 - 0.608) |
| No | Female | heavy | Spring | 0.701 (0.601 - 0.789) | |
| No | Male | light | Summer | 0.222 (0.122 - 0.345) | |
| No | Male | light | Autumn | 0.684 (0.604 - 0.757) | |
| No | Male | light | Winter | 0.743 (0.638 - 0.829) | 0.497 (0.354 - 0.636) |
| No | Male | light | Spring | 0.723 (0.623 - 0.808) | |
| No | Male | average | Summer | 0.523 (0.444 - 0.605) | |
| No | Male | average | Autumn | 0.684 (0.604 - 0.757) | |
| No | Male | average | Winter | 0.743 (0.638 - 0.829) | 0.497 (0.354 - 0.636) |
| No | Male | average | Spring | 0.723 (0.623 - 0.808) | |
| No | Male | heavy | Summer | 0.771 (0.652 - 0.858) | |
| No | Male | heavy | Autumn | 0.684 (0.604 - 0.757) | |
| No | Male | heavy | Winter | 0.743 (0.638 - 0.829) | 0.497 (0.354 - 0.636) |
| No | Male | heavy | Spring | 0.723 (0.623 - 0.808) | |
| Yes | Female | light | Summer | 0.355 (0.192 - 0.544) | |
| Yes | Female | light | Autumn | 0.661 (0.586 - 0.732) | |
| Yes | Female | light | Winter | 0.723 (0.615 - 0.812) | 0.465 (0.324 - 0.608) |
| Yes | Female | light | Spring | 0.701 (0.601 - 0.789) | |
| Yes | Female | average | Summer | 0.652 (0.554 - 0.742) | |
| Yes | Female | average | Autumn | 0.661 (0.586 - 0.732) | |
| Yes | Female | average | Winter | 0.723 (0.615 - 0.812) | 0.465 (0.324 - 0.608) |
| Yes | Female | average | Spring | 0.701 (0.601 - 0.789) | |
| Yes | Female | heavy | Summer | 0.845 (0.767 - 0.901) | |
| Yes | Female | heavy | Autumn | 0.661 (0.586 - 0.732) | |
| Yes | Female | heavy | Winter | 0.723 (0.615 - 0.812) | 0.465 (0.324 - 0.608) |
| Yes | Female | heavy | Spring | 0.701 (0.601 - 0.789) | |
| Yes | Male | light | Summer | 0.387 (0.214 - 0.568) | |
| Yes | Male | light | Autumn | 0.684 (0.604 - 0.757) | |
| Yes | Male | light | Winter | 0.743 (0.638 - 0.829) | 0.497 (0.354 - 0.636) |
| Yes | Male | light | Spring | 0.723 (0.623 - 0.808) | |
| Yes | Male | average | Summer | 0.676 (0.579 - 0.766) | |
| Yes | Male | average | Autumn | 0.684 (0.604 - 0.757) | |
| Yes | Male | average | Winter | 0.743 (0.638 - 0.829) | 0.497 (0.354 - 0.636) |
| Yes | Male | average | Spring | 0.723 (0.623 - 0.808) | |
| Yes | Male | heavy | Summer | 0.857 (0.781 - 0.912) | |
| Yes | Male | heavy | Autumn | 0.684 (0.604 - 0.757) | |
| Yes | Male | heavy | Winter | 0.743 (0.638 - 0.829) | 0.497 (0.354 - 0.636) |
| Yes | Male | heavy | Spring | 0.723 (0.623 - 0.808) | |

Code S1: JAGS model code for the Cormack-Jolly-Seber model estimating biweekly survival and resighting frequency of radio-tracked little owls in Germany between 2009 – 2011. Data and R code to run this model are available at <https://github.com/Vogelwarte/LittleOwlSurvival>

model {

# Priors and constraints

for (i in 1:nind){

for (t in f[i]:(n.occasions)){

logit(phi[i,t]) <- mu[season[t]] +

beta.mass*weight[i]*pf[t] +

beta.feed*feeding[i]*pf[t] +

beta.win*env[year[i],t] +

beta.male*sex[i]

logit(p[i,t]) <- mu.p[recap.mat[i,t]] + beta.p.win*env[year[i],t] + epsilon.p[i]

} #t

} #i

for (i in 1:nind){

epsilon.p[i] ~ dnorm(0, tau.p)

}

for (s in 1:4){ ### baseline for the 4 seasons summer, autumn, winter, spring

mean.phi[s] ~ dbeta(95, 10) # Prior for mean biweekly survival from Thorup et al. 2013, converted to beta

mu[s] <- log(mean.phi[s] / (1-mean.phi[s])) # Logit transformation

}

mean.p[1] ~ dunif(0.7, 1) # Prior for mean recapture during full effort periods

mean.p[2] ~ dunif(0.3, 0.9) # Prior for mean recapture during reduced effort periods

for (y in 1:2) {

mu.p[y] <- log(mean.p[y] / (1-mean.p[y])) # Logit transformation

}

mu.p[3] <- -999999999999999999 # recapture probability of zero on logit scale

sigma.p ~ dunif(0, 2) # Prior for standard deviation for random detection effect

tau.p <- pow(sigma.p, -2)

beta.mass ~ dnorm(0, 1) # Prior for mass effect

beta.male ~ dnorm(0, 1) # Prior for sex effect (for males, females are 0)

beta.win ~ dunif(-2, 2) # Prior for winter weather effect, which we suspect is negative

beta.p.win ~ dnorm(0, 1) # Prior for winter weather DETECTION effect

beta.feed ~ dnorm(0, 1) # Prior for effect of supplementary feeding

# Likelihood

for (i in 1:nind){

# Define latent state at first capture

z[i,f[i]] <- 1

z.rep[i,f[i]] <- 1 # replicate z (true state)

y.rep[i,f[i]] <- 1 # replicate y (data)

for (t in (f[i]+1):n.occasions){

# State process

z[i,t] ~ dbern(phi[i,t-1] * z[i,t-1])

z.rep[i,t] ~ dbern(phi[i,t-1] * z.rep[i,t-1]) # replicate z (true state)

# Observation process

y[i,t] ~ dbern(p[i,t] * z[i,t])

y.rep[i,t] ~ dbern(p[i,t] * z.rep[i,t]) # replicate y (observations)

} #t end

#Derived parameters

## GOODNESS OF FIT TEST SECTION

## Discrepancy observed data

E.obs[i] <- pow((sum(y[i,(f[i]+1):n.occasions]) - sum(p[i,(f[i]+1):(n.occasions)] * z[i,(f[i]+1):n.occasions])), 2) / (sum(p[i,(f[i]+1):n.occasions] * z[i,(f[i]+1):n.occasions]) + 0.001)

## Discrepancy replicated data

E.rep[i] <- pow((sum(y.rep[i,(f[i]+1):n.occasions]) - sum(p[i,(f[i]+1):(n.occasions)] * z.rep[i,(f[i]+1):n.occasions])), 2) / (sum(p[i,(f[i]+1):(n.occasions)] * z.rep[i,(f[i]+1):n.occasions]) + 0.001)

} #i end

fit <- sum(E.obs[])

fit.rep <- sum(E.rep[])

}
